# Comparison of functional aspects and quality of life before and after using the heat and moisture exchange in total laryngectomy patients

**DOI:** 10.1590/2317-1782/e20240282en

**Published:** 2025-10-17

**Authors:** Romulo da Silva Rodrigues, Victória Paes Leme da Silva, Leticia Cristina de Jesus dos Santos, Guilherme Maia Zica, Mariana Pinheiro Brendim

**Affiliations:** 1 Departamento de Fonoaudiologia, Universidade Federal do Rio de Janeiro – UFRJ - Rio de Janeiro (RJ), Brasil.; 2 Programa de Pós-graduação em Distúrbios da Comunicação Humana, Universidade Federal de São Paulo – UNIFESP - São Paulo (SP), Brasil.

**Keywords:** Laryngectomy, Tracheostomy, Quality of Life, Respiration, Cough, Voice, Deglutition, Sleep Quality, Speech-Language Pathology

## Abstract

**Purpose:**

To compare self-assessment of voice handicap, sleep quality, and quality of life related to coughing and swallowing before and after using a heat and moisture exchange filter in total laryngectomized patients.

**Methods:**

This was a prospective longitudinal study whose participants completed the Voice Handicap Index (VHI), Pittsburgh Sleep Quality Index (PSQI), Leicester Cough Questionnaire (LCQ), and MD Anderson Dysphagia Inventory (MDADI) at three times: (T1) before starting to use the heat and moisture exchange filter, (T2) 2 weeks after using it, and (T3) 4 weeks after using it.

**Results:**

14 participants (12 men) with a mean age of 66.4 ± 5.8 years. The medians of the total VHI score at T1, T2, and T3 were, respectively, 65.5 (47.5-86.3), 55.5 (39.5-71.3), and 44.5 (39-72), p=0.085. The medians of the PSQI score at T1, T2, and T3 were, respectively, 6.5 (4.25-11.8), 4.5 (2.25-10.8), and 3.0 (2.0-5.75), p=0.010. The medians of the total MDADI score at T1, T2, and T3 were, respectively, 78.6 (69.1-92.7), 76.3 (73.3-92.6), and 85.7 (72.7-94), p=0.571. The medians of the total LCQ score at T1, T2, and T3 were, respectively, 16.7 (13.1-18.5), 19.1 (17.4-19.4), and 19.0 (17.3-19.9), p=0.002.

**Conclusion:**

The total laryngectomized patients participating in this study self-assessed an improvement in the emotional domain of voice handicap, sleep quality, and cough-related quality of life after 2 weeks of using a heat and moisture exchange filter. They also maintained the perception of improvement after 4 weeks of using the device.

## INTRODUCTION

Total laryngectomy (TL), considered the primary treatment for advanced laryngeal carcinoma, is an aggressive surgical procedure with negative and long-lasting functional and aesthetic results^(1-3)^. The literature reports a great impact on the patient's quality of life due to the impairment and modifications of essential functions for individuals, such as voice, swallowing, smell, taste, and breathing^(1)^.

Physiological changes after total LT are due to the removal of several structures, the separation of the respiratory and digestive tracts, and the construction of a permanent stoma. Specific changes in the voice and respiratory systems are caused by the dissociation of the upper and lower airways, preventing the natural production of laryngeal voice and interrupting the normal pathway of air preparation through the nose^(4-6)^. Little is known about the swallowing process after total LT. Manometric studies of swallowing have observed an increase in the duration of velopharyngeal pressure and a decrease in upper esophageal sphincter pressure, demonstrating the effects of performing cricopharyngeal myotomy and rupturing the cricopharyngeal and rostral esophageal muscle fibers from their attachments to the larynx^(6)^.

The stoma allows unconditioned airflow directly to the lower airways (trachea). Therefore, it prevents the maintenance of heating, humidification, filtration, and physiological resistance of inhaled air and generates a deficit in olfactory function. The entry of cold, dry air, microorganisms, and dust directly into the lower airways increases the incidence of bronchopulmonary damage and infections and reduces quality of life^(1,4,7)^.

Due to most TL patients’ history of smoking, the post-laryngectomy state may impair lung function and ventilation (gas exchange). Tracheostomized patients experience reduced aerodynamic airflow resistance during inspiration and expiration due to direct inhalation through the stoma. This can negatively affect peripheral ventilation and favor alveolar collapse^(8,9)^.

One of the most important prognostic factors in the survival of laryngectomized patients is the progressive deterioration of lung function^(10,11)^. Nevertheless, few studies in the literature have evaluated lung function and its integrity in such patients or described rehabilitation and its effects using a reproducible methodology^(4,11-14)^. According to one study, most patients undergoing TL have obstructive abnormal lung function, almost always associated with a history of smoking^(11)^.

Quality of life is a complex, multifaceted concept, dependent on the person's perspective of physical, psychological, and functional health, as well as social and financial well-being^(15-17)^. TL surgery is disfiguring and requires a permanent stoma, which is potentially stigmatizing and alters communication and intimate relationships, with difficult-to-manage complications such as excess pulmonary secretion, recurrent cough, and sleep difficulties^(1,4,7,12,15)^. Furthermore, postoperative social integration has been shown to be deficient, with depression, anxiety, and self-isolation^(1,18)^. These aspects reaffirm the need for a multidimensional investigation of each patient’s perceptions, their position in life within the culture and value systems in which they live, and their goals, expectations, standards, and concerns.

Various respiratory complaints are importantly correlated with individuals' physical and psychosocial problems. Rehabilitation typically focuses more on voice and, sometimes, swallowing and smell^(5,6)^. Thus, it is believed that a complete speech-language-hearing rehabilitation program for TL patients should consider the management of respiratory deficits resulting from the surgical procedure^(4,19)^.

A viable option for post-TL pulmonary rehabilitation is the heat and moisture exchanger (HME), placed over a hermetic seal around the tracheal stoma. The HME has three physical properties: heat and moisture exchange capacity, added airflow resistance, and particle filtration compatible with nasal function. There are different adhesives (stoma seal and fixation base), with distinct adhesion and adaptation properties to facilitate functionality for each patient and each stoma’s anatomical variations^(4,12-14)^.

Clinical experience and various studies have shown a noticeable reduction in coughing and mucus production among HME users who have undergone TL^(4,19)^. However, particularly in Brazil and Latin America, scientific evidence demonstrating the benefits of this device and the importance of its use is still limited.

Hence, this study aimed to compare the self-assessment of voice handicap, sleep quality, and quality of life related to coughing and swallowing before and after using an HME filter in TL patients at a university hospital in Brazil.

## METHOD

This study was approved by the Research Ethics Committee of the Clementino Fraga Filho University Hospital (HUCFF), under approval number 3,442,414. All participants signed an informed consent form.

This is an uncontrolled trial conducted between August 2019 and May 2022 at HUCFF’s speech-language-hearing outpatient clinic. Inclusion criteria were adults undergoing TL surgery and receiving speech-language-hearing treatment at this hospital. Exclusion criteria were individuals with neurological diseases, cognitive or language impairments, undergoing end-of-life care, and already using a tracheostomy humidifier filter.

Participants were recruited consecutively, including all TL individuals undergoing speech-language-hearing treatment at this hospital during the study period. The sample consisted of 16 men and two women undergoing TL surgery, with a mean age of 66.2 ± 5.3 years.

Individuals who started the study and discontinued follow-up for any reason during the study procedure were considered lost to follow-up. Therefore, only those who completed all study stages without interruption remained in the study.

The study applied the Brazilian version of the Voice Handicap Index (VHI)^(20)^, MD Anderson Dysphagia Inventory (MDADI)^(21)^, Leicester Cough Questionnaire (LCQ)^(22)^, and Pittsburgh Sleep Quality Index (PSQI)^(23)^ to all participants three times: (T1) before starting to use the HME filter, (T2) after 2 weeks of using the HME filter, and (T3) after 4 weeks of using the HME filter.

The VHI^(24)^, adapted and validated for Brazilian Portuguese, has 30 items, whose response options cover three domains: functional, physical, and emotional^(20)^. The score for each domain ranges from 0 to 40, and the total score ranges from 0 to 120 points. The higher the score, the greater the perception of voice handicap^(20)^.

The MDADI^(25)^, adapted and validated for Brazilian Portuguese, assesses dysphagia-related quality of life specifically in the population with head and neck cancer^(21)^. Its 20 items cover the global issue and the physical, functional, and emotional domains, with a range of up to 100 points. The lower the score, the greater the impact on quality of life^(21)^.

The LCQ^(26)^, translated and adapted into Brazilian Portuguese, assesses the quality of life in individuals with cough. It consists of 19 items, comprising the physical, psychological, and social domains. The score for each domain ranges from 1 to 7 points, and the total score ranges from 3 to 21 – scores close to 21 indicate better health status or less influence of cough on quality of life^(22)^.

The PSQI^(27)^, translated and validated into Brazilian Portuguese, assesses sleep quality and disorders. Its score ranges up to 21 points; those above 5 indicate poor sleep quality^(23)^.

The participants’ sociodemographic and clinical information was also collected, including sex; age; time since laryngectomy, including the period between the date of surgery and the date of entry into the study; whether disease treatment included neck dissection, radiotherapy, or chemotherapy; presence or absence of lung disease and tracheostomy cannula; whether tracheostomy was performed as an emergency (before TL) or during the surgery; history of smoking; and laryngeal communication methods used.

Weekly meetings were held at the HUCFF speech-language-hearing outpatient clinic to adapt the HME filter. Participants received seven Provox^®^ XtraFlow™ HME filters and seven Provox^®^ Adhesive OptiDerm™ oval patches for 4 consecutive weeks. They were instructed to perform local hygiene, then adhere the patch to clean skin at the tracheostoma level and adapt the filter to the adhesive holder. Those using a tracheostomy tube were instructed to adapt the filter to the Provox^®^ LaryTube™. All participants were instructed to wear the filter full-time, removing it only to change it every 24 hours or to clean the tracheostomy tube.

During the 4 weeks of device use, participants did not undergo speech-language-hearing therapy for swallowing, manual lymphatic drainage, or myofascial release. This study did not instruct them to use inhalation. None of the participants underwent respiratory physiotherapy.

Statistical analysis was performed using Jamovi version 1.6.23. Data normality was verified using a histogram. The T1, T2, and T3 scores were compared to verify self-assessment before and after using the tracheostomy humidifier filter. Questionnaire scores were compared using Friedman's ANOVA test, followed by the Durbin-Conover multiple comparison test. The level of statistical significance was set at 5%.

## RESULTS

**Figure 1:**
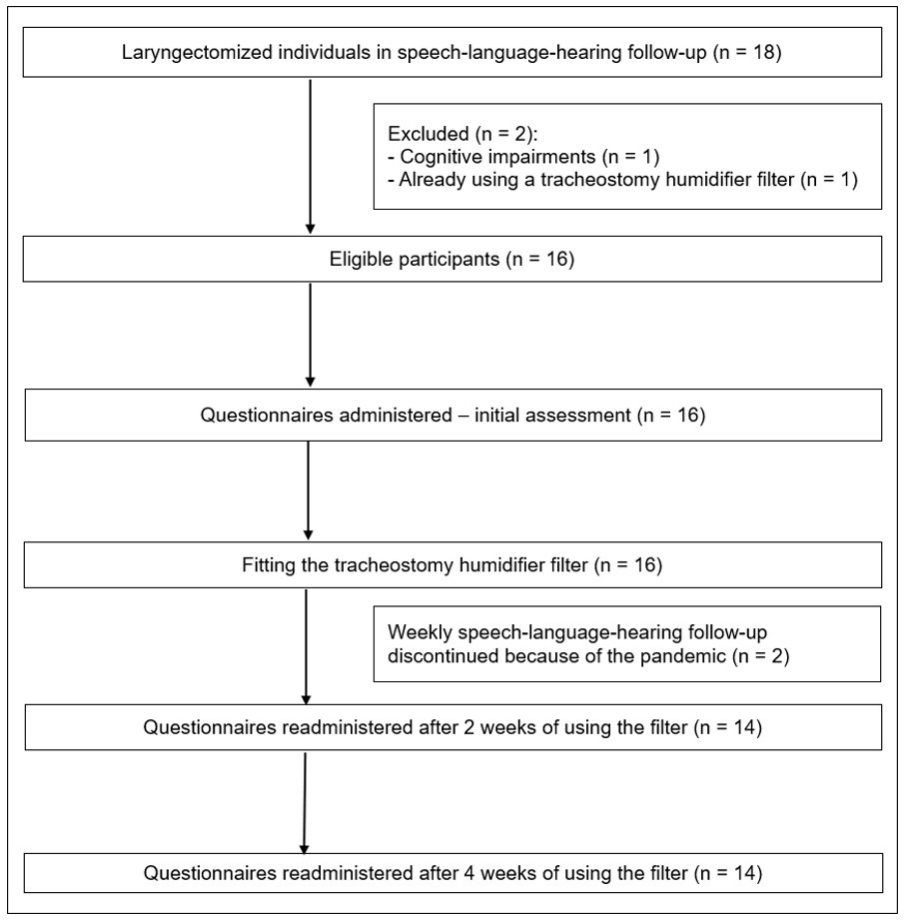
Flowchart of study participants

The flowchart of study participants is shown in Figure 1. Two of the 18 study participants were excluded, and two were lost to follow-up, totaling 14 participants in the final sample.

The final sample comprised 12 men and two women, aged 60 to 81 years, with a mean age of 66.4±5.8 years. Their characteristics are presented in Table 1. Those with lung disease had chronic obstructive pulmonary disease. Regarding the communication method, two (14.3%) participants used the tracheoesophageal voice, and 12 (85.7%) used the esophageal voice; four participants were fluent, and the others were learning.

**Table 1:** Characteristics of participants

**Characteristics**	**Total (n = 14)**
Males, n (%)	12 (85.7%)
Age (years), median (IQR)	66 (61.5-69)
Time since laryngectomy (months), median (IQR)	15 (8.5-29.3)
Neck dissection, n (%)	13 (92.9%)
Radiotherapy, n (%)	12 (85.7%)
Chemotherapy, n (%)	6 (42.9%)
History of smoking, n (%)	12 (85.7%)
Pulmonary disease, n (%)	4 (28.6%)
Emergency tracheostomy, n (%)	5 (35.7%)
Use of tracheostomy cannula (LaryTube)	1 (7.1%)

Caption: IQR = interquartile range

Comparisons of the VHI total, physical, functional, and emotional scores are presented in Table 2. There was a statistically significant difference in the emotional domain scores (*x* ^2^ (2) = 10.1, p = 0.006). Post hoc analysis indicated statistically significant differences in the emotional domain scores between T1 and T2 (p = 0.022) and T1 and T3 (p = 0.001), but not between T2 and T3 (p = 0.192).

**Table 2:** Comparison of the Voice Handicap Index between moments T1, T2, and T3

**VHI**	**T1**	**T2**	**T3**	**p-value**
Physical domain	27(21.3-30.8)	24.5(21.3-30.8)	25(16.5-30.3)	0.651
Functional domain	20.5(11.3-24.5)	15(8.75-23.3)	12.5(9.5-20.8)	0.614
Emotional domain	22.5(7.25-32)	14.5(5.75-23.5)^a^	14(4.5-18.8)^b^	**0.006**
Total score	65.5(47.5-86.3)	55.5(39.5-71.3)	44.5(39-72)	0.085

Friedman test. Pairwise comparison using the Durbin-Conover test:

a p = 0.022 when comparing T1 and T2;

b p < 0.001 when comparing T1 and T3

Values ​​are presented as median and interquartile range

Caption: VHI = Voice Handicap Index; T1 = initial assessment; T2 = assessment after 2 weeks; T3 = assessment after 4 weeks

Comparisons of the MDADI total, global, emotional, functional, and physical scores are presented in Table 3. There was no statistically significant difference in any of the MDADI scores.

**Table 3:** Comparison of the MD Anderson Dysphagia Inventory between moments T1, T2, and T3

**MDADI**	**T1**	**T2**	**T3**	**p-value**
Global	70(40-80)	80(45-100)	100(50-100)	0.087
Emotional domain	80(73.3-100)	81.7(69.2-100)	91.7(77.5-100)	0.590
Functional domain	84(80-95)	86(80-96)	94(78-96)	0.832
Physical domain	71.3(65-80)	76.3(65-81.9)	68.8(65-84.4)	0.853
Total score	78.6(69.1-92.7)	76.3(73.3-92.6)	85.7(72.7-94.0)	0.571

Friedman test

Values ​​are presented as median and interquartile range

Caption: MDADI = MD Anderson Dysphagia Inventory; T1 = initial assessment; T2 = assessment after 2 weeks; T3 = assessment after 4 weeks

Comparisons of the LCQ total, physical, psychological, and social scores are presented in Table 4. There were statistically significant differences in its total scores (*x* ^2^ (2) = 12.7, p = 0.002), physical domain (*x* ^2^ (2) = 9.16, p = 0.010), psychological domain (*x* ^2^ (2) = 10.1, p = 0.006), and social domain (*x* ^2^ (2) = 9.94, p = 0.007) between the assessment times. Post hoc analysis indicated statistically significant differences in the LCQ total scores (p = 0.003) and the physical (p = 0.032) and psychological (p = 0.016) domains between T1 and T2. Furthermore, post hoc analysis indicated statistically significant differences in total scores (p < 0.001) and all domains (physical, p = 0.039; psychological, p = 0.004; and social, p = 0.02) between T1 and T3. There was no statistically significant difference in any score between T2 and T3.

**Table 4:** Comparison of the Leicester Cough Questionnaire between moments T1, T2, and T3

**LCQ**	**T1**	**T2**	**T3**	**p-value**
Physical domain	4.88(3.81-5.60)	6.13(5.44-6.25)^a^	6.13(5.25-6.44)^b^	**0.010**
Psychological domain	5.29(4.33-6.10)	6.14(5.43-6.57)^a^	6.14(5.82-6.60)^b^	**0.006**
Social domain	6.25(5.31-7.00)	6.88(6.19-7.00)	7.00(6.19-7.00)^b^	**0.007**
Total score	16.7(13.1-18.5)	19.1(17.4-19.4)^a^	19.0(17.3-19.9)^b^	**0.002**

Friedman test. Pairwise comparison using the Durbin-Conover test:

a p < 0.05 in the comparison between T1 and T2;

b p < 0.05 in the comparison between T1 and T3

Values ​​are presented as median and interquartile range

Caption: LCQ = Leicester Cough Questionnaire; T1 = initial assessment; T2 = assessment after 2 weeks; T3 = assessment after 4 weeks

Comparisons of PSQI component scores are presented in Table 5. There was a statistically significant difference in the total PSQI score between assessment times (*x* ^2^ (2) = 9.23, p = 0.010). Post hoc analysis indicated statistically significant differences in scores between T1 and T2 (p = 0.029) and T1 and T3 (p = 0.002), but not between T2 and T3 (p = 0.236). There was also a statistically significant difference in sleep disturbance scores between assessment times (*x* ^2^ (2) = 9.00, p = 0.011). Post hoc analysis indicated statistically significant differences in the scores of this component between T1 and T3 (p = 0.002), but not between T1 and T2 (p = 0.091) or between T2 and T3 (p = 0.091).

**Table 5:** Comparison of sleep quality between moments T1, T2, and T3

**PSQI components**	**T1**	**T2**	**T3**	**p-value**
Subjective sleep quality	1(1-2)	1(0-1.75)	1(0-1)	0.054
Sleep latency	1(1-1)	1(1-1.75)	1(0-1)	0.661
Sleep duration	1(0.25-1)	0.5(0-2)	0(0-1)	0.069
Habitual sleep efficiency	1 (0-2)	1(0-2)	0(0-1.75)	0.459
Sleep disturbances	2(1-2)	1(1-2)	1(1-1)^b^	**0.011**
Use of sleep medication	0(0-0.75)	0(0-0)	0(0-0)	0.150
Daytime dysfunction	1(0-1.75)	0(0-1)	0(0-1)	0.597
Total score	6.50(4.25-11.8)	4.50(2.25-10.8)^a^	3(2.0-5.75)^b^	**0.010**

Friedman test. Pairwise comparison using the Durbin-Conover test:

a p < 0.05 in the comparison between T1 and T2;

b p < 0.05 in the comparison between T1 and T3

Values ​​are presented as median and interquartile range

Caption: PSQI = Pittsburgh Sleep Quality Index

## DISCUSSION

This appears to be the first study to use validated self-assessment instruments to investigate the perceptions of Brazilian TL patients regarding vocal aspects, swallowing, and sleep, before and after using an HME filter. It also investigated self-assessment of cough-related quality of life, using an instrument translated and adapted into Brazilian Portuguese, before and after using an HME filter. The study results demonstrate general improvements in aspects related to the perception of voice handicap, cough-related quality of life, and sleep quality after using an HME filter.

In line with the literature, the study participants’ profile was predominantly older male individuals with a history of smoking, since laryngeal cancer is more frequent in men, with a mean age over 60 years, with smoking being its main risk factor^(28-30)^.

Regarding the perception of voice handicap, the median total VHI score in this study, in which most participants used esophageal voice, was similar to the mean total VHI score reported by other researchers in individuals with esophageal voice^(31)^.

Although our results revealed a reduction in the VHI total, physical, functional, and emotional scores over the 4 weeks of HME filter use, only the emotional domain had a statistically significant change both at 2 and 4 weeks of use. This means they perceived an improvement in emotional aspects after 2 weeks of HME filter use, and that this perception of improvement is maintained after 4 weeks of using the device. This instrument’s emotional domain includes items related to frustration in general communication situations and the perception of handicaps due to voice changes, embarrassment when repeating statements, feelings of incompetence, and shame^(32)^.

In line with our results, a study that evaluated the responses of Brazilian TL patients to the question, “What do you think of your voice?” revealed an improvement in vocal self-perception in 70% of individuals after 2 weeks of using an HME filter^(4)^. In contrast, another study investigated the auditory-perceptual evaluation of the voice of Brazilian laryngectomees and found no influence of the use of an HME filter for 6 weeks on esophageal or tracheoesophageal voice quality^(12)^.

Regarding swallowing-related quality of life, the study participants’ MDADI scores were similar to those reported in the literature^(28,29)^. Furthermore, according to the proposal by Chen et al.^(33)^, the median total MDADI score found in this study indicates an average limitation in these individuals’ swallowing quality of life.

This study observed no statistically significant changes in the MDADI total, global, emotional, functional, or physical scores after using the HME filter. This indicates that the study participants perceived neither improvement nor worsening in their swallowing-related quality of life, either after 2 or 4 weeks of using the device. It is believed that swallowing in patients undergoing TL requires focused and specialized intervention to control and overcome motor and psycho-emotional deficits.

The study results show that TL individuals improved in all cough-related quality-of-life domains after 4 weeks of using an HME filter. Unconditioned air flows directly to the epithelium of the lower respiratory tract after surgery, due to anatomical changes and redirection of respiratory flow via tracheostomy. This potentially causes histological changes in the tracheobronchial mucosa, excessive secretion production, recurrent involuntary coughing, and forced expectoration to clear the airways of mucus^(34,35)^. Thus, the results regarding the improvement in cough-related quality of life in this study can be explained by the effect of the device on filtering, heating, and humidifying the air inhaled via the tracheostomy. The physical, psychological, and total scores improved significantly after 2 weeks of using the HME filter, but not the social domain of quality of life. The results agree with other studies, which also found improvements in self-perception of cough, cough frequency, and overall quality-of-life index after 2 weeks of using the HME filter^(4,13)^.

One possible explanation for the social domain only improving after 4 weeks of filter use is that social functioning depends on social interaction and speech skills, which take longer to develop in TL patients who use esophageal voice during rehabilitation. Other studies have found reduced social isolation and increased frequency of social interaction among those using the HME filter, due to the greater ease of communication, better social interaction, and less embarrassment related to the production and expectoration of secretions^(18,36)^.

Regarding sleep quality, our results show that the median total PSQI score was greater than 5 before using the HME filter, indicating that at least half of the participants had poor sleep quality. This aspect is rarely considered in post-TL rehabilitation and management.

After 2 weeks of using the filter, participants improved their perception of sleep quality significantly, which continued after 4 weeks of use. A meta-analysis study showed that the HME filter causes fewer sleep problems after TL than an external humidifier^(37)^. Furthermore, median total PSQI scores below 5 after 2 and 4 weeks of using the HME filter indicate that at least half of the participants in this study no longer perceived poor sleep quality, unlike their perception before using the device.

These people’s perceived improvement in sleep quality may be explained by their possibly experiencing greater comfort when breathing and a lower risk of awakening during sleep, due to the reduction in secretion and, consequently, coughing after using the device. Furthermore, the safety of not having an open orifice during the night may allow for greater comfort and relaxation during sleep.

It should be emphasized that this study has some important limitations. The first is the lack of a control group (one not using the HME filter) for comparison with the group using the device. Therefore, this study does not allow us to state that the observed changes were due to the use of the HME filter, as it is not a randomized clinical trial. The second limitation concerns the sample size, with few participants, leading to the possibility of type II error and the impossibility of generalizing the results. Another limitation is the instruments used. The MDADI, although validated in our language for assessing swallowing-related quality of life in the head and neck cancer population, was not developed specifically for TL patients^(38)^. Furthermore, the LCQ, although translated and adapted to Brazilian Portuguese and assessing cough-related quality of life, has not completed the validation stages.

## CONCLUSION

TL patients in this study reported perceived improvements in the emotional domain of self-assessed voice handicap, sleep quality, and cough-related quality of life after 2 weeks of using an HME filter. The improvement perceived in these aspects was maintained after 4 weeks of device use, although there was no perceived improvement in swallowing quality of life after 2 or 4 weeks of device use among study participants.

## References

[1] Scott AJ, McGuire JK, Manning K, Leach L, Fagan JJ (2019). Quality of life after total laryngectomy: evaluating the effect of socioeconomic status. J Laryngol Otol.

[2] Stokes WA, Jones BL, Bhatia S, Oweida AJ, Bowles DW, Raben D (2017). A comparison of overall survival for patients with T4 larynx cancer treated with surgical versus organ‐preservation approaches: a national cancer data base analysis. Cancer.

[3] Barbosa LNF, Francisco ALP (2011). Paciente laringectomizado total: perspectivas para a ação clínica do psicólogo. Paideia..

[4] Araujo AMBD, Santos ECBD, Pernambuco L (2017). Breathing and voice self-assessments after the use of a heat and moisture exchange in total laryngectomyzed patients. Audiol Commun Res.

[5] Rosa VM, Fores JML, Silva EPF, Guterres EO, Marcelino A, Nogueira PC (2018). Interdisciplinary interventions in the perioperative rehabilitation of total laryngectomy: an integrative review. Clinics.

[6] Lippert D, Hoffman MR, Britt CJ, Jones CA, Hernandez J, Ciucci MR (2016). Preliminary evaluation of functional swallow after total laryngectomy using high-resolution manometry. Ann Otol Rhinol Laryngol.

[7] Mérol JC, Charpiot A, Langagne T, Hémar P, Ackerstaff AH, Hilgers FJ (2012). Randomized controlled trial on postoperative pulmonary humidification after total laryngectomy: external humidifier versus heat and moisture exchanger. Laryngoscope.

[8] Heyden R (1950). The respiratory function in laryngectomized patients. Acta Otolaryngol.

[9] Torjussen W (1968). Airway obstructions in laryngectomized patients: a spirometric investigation. Acta Otolaryngol.

[10] Todisco T, Maurizi M, Paludetti G, Dottorini M, Merante F (1984). Laryngeal cancer: long-term follow-up of respiratory functions after laryngectomy. Respiration.

[11] Castro MA, Dedivitis RA, Salge JM, Matos LL, Cernea CR (2019). Evaluation of lung function in patients submitted to total laryngectomy. Rev Bras Otorrinolaringol.

[12] Masson ACC, Fouquet ML, Gonçalves AJ (2008). Umidificador de traqueostoma: influência na secreção e voz de laringectomizados. Pro Fono.

[13] Parrilla C, Minni A, Bogaardt H, Macri GF, Battista M, Roukos R (2015). Pulmonary rehabilitation after total laryngectomy: a multicenter time-series clinical trial evaluating the Provox XtraHME in HME-Naïve patients. Ann Otol Rhinol Laryngol.

[14] Herranz J, Espiño MA, Morado CO (2013). Pulmonary rehabilitation after total laryngectomy: a randomized cross-over clinical trial comparing two different heat and moisture exchangers (HMEs). Eur Arch Otorhinolaryngol.

[15] Hoffman HT, Porter K, Karnell LH, Cooper JS, Weber RS, Langer CJ (2006). Laryngeal cancer in the United States: changes in demographics, patterns of care, and survival. Laryngoscope.

[16] Murphy B, Herrman H, Hawthorne G, Pinzone T, Evert H (2000). Australian WHOQoL instruments: user’s manual and interpretation guide..

[17] Perry A, Casey E, Cotton S (2015). Quality of life after total laryngectomy: functioning, psychological well-being and self-efficacy. Int J Lang Commun Disord.

[18] Brook I, Bogaardt H, van As-Brooks C (2013). Long-term use of heat and moisture exchangers among laryngectomees: medical, social, and psychological patterns. Ann Otol Rhinol Laryngol.

[19] Icuspit P, Yarlagadda B, Garg S, Johnson T, Deschler D (2014). Heat and moisture exchange devices for patients undergoing total laryngectomy. ORL Head Neck Nurs.

[20] Behlau M, Santos LMA, Oliveira G (2011). Cross-cultural adaptation and validation of the voice handicap index into Brazilian Portuguese. J Voice.

[21] Guedes RLV, Angelis EC, Chen AY, Kowalski LP, Vartanian JG (2013). Validation and application of the M.D. Anderson Dysphagia Inventory in patients treated for head and neck cancer in Brazil. Dysphagia.

[22] Felisbino MB, Steidle LJM, Gonçalves-Tavares M, Pizzichini MMM, Pizzichini E (2014). Leicester Cough Questionnaire: translation to Portuguese and cross-cultural adaptation for use in Brazil. J Bras Pneumol.

[23] Bertolazi AN, Fagondes SC, Hoff LS, Dartora EG, Miozzo IC, de Barba ME (2011). Validation of the Brazilian Portuguese version of the Pittsburgh Sleep Quality Index. Sleep Med.

[24] Jacobson BH, Johnson A, Grywalski C, Silbergleit A, Jacobson G, Benninger MS (1997). The Voice Handicap Index (VHI): development and validation. Am J Speech Lang Pathol.

[25] Chen AY, Frankowski R, Bishop-Leone J, Hebert T, Leyk S, Lewin J (2001). The development and validation of a dysphagia-specific quality-of-life questionnaire for patients with head and neck cancer: the M. D. Anderson dysphagia inventory. Arch Otolaryngol Head Neck Surg.

[26] Birring SS, Prudon B, Carr AJ, Singh SJ, Morgan MD, Pavord ID (2003). Development of a symptom specific health status measure for patients with chronic cough: Leicester Cough Questionnaire (LCQ). Thorax.

[27] Buysse DJ, Reynolds CF, Monk TH, Berman SR, Kupfer DJ (1989). The Pittsburgh Sleep Quality Index: a new instrument for psychiatric practice and research. Psychiatry Res.

[28] Kemps GJF, Krebbers I, Pilz W, Vanbelle S, Baijens LWJ (2020). Affective symptoms and swallow-specific quality of life in total laryngectomy patients. Head Neck.

[29] Wulff NB, Dalton SO, Wessel I, Arenaz Búa B, Löfhede H, Hammerlid E (2022). Health-Related Quality of Life, Dysphagia, Voice Problems, Depression, and Anxiety After Total Laryngectomy. Laryngoscope.

[30] Cîrstea AI, Berteșteanu ȘVG, Scăunașu RV, Popescu B, Bejenaru PL, Simion-Antonie CB (2023). Management of locally advanced laryngeal cancer-from risk factors to treatment, the experience of a tertiary hospital from Eastern Europe. Int J Environ Res Public Health.

[31] Dragičević D, Jović RM, Kljajić V, Vlaški L, Savović S (2020). Comparison of voice handicap index in patients with esophageal and tracheoesophageal speech after total laryngectomy. Folia Phoniatr Logop.

[32] Rosa ME, Mituuti CT, Ghirardi ACAM (2018). Correlation between the Voice Handicap and Swallowing Quality of Life in patients with laryngeal cancer submitted to chemoradiotherapy. CoDAS.

[33] Chen PH, Golub JS, Hapner ER, Johns MM (2009). Prevalence of perceived dysphagia and quality-of-life impairment in a geriatric population. Dysphagia.

[34] Ward EC, Hancock K, Boxall J, Burns CL, Spurgin AL, Lehn B (2023). Post-laryngectomy pulmonary and related symptom changes following adoption of an optimal day-and-night heat and moisture exchanger (HME) regimen. Head Neck.

[35] Ebersole B, Moran K, Gou J, Ridge J, Schiech L, Liu JC (2020). Heat and moisture exchanger cassettes: results of a quality/safety initiative to reduce postoperative mucus plugging after total laryngectomy. Head Neck.

[36] Lydiatt WM, Moran J, Burke WJ (2009). A review of depression in the head and neck cancer patient. Clin Adv Hematol Oncol H&O..

[37] Tawfik GM, Makram OM, Zayan AH, Ghozy S, Eid PS, Mahmoud MH (2021). Voice rehabilitation by voice prostheses after total laryngectomy: a systematic review and network meta-analysis for 11,918 patients. J Speech Lang Hear Res.

[38] Govender R, Lee MT, Davies TC, Twinn CE, Katsoulis KL, Payten CL (2012). Development and preliminary validation of a patient-reported outcome measure for swallowing after total laryngectomy (SOAL questionnaire). Clin Otolaryngol.

