# Supplementary material for: Comparison of functional aspects and quality of life before and after using the heat and moisture exchange in total laryngectomy patients
Source: Codas. 2025 Oct 17;37(5):e20240282. doi: 10.1590/2317-1782/e20240282en (PMC12533531; doi:10.1590/2317-1782/e20240282en)
Supplement: Translation

# Translation: Comparação dos aspectos funcionais e qualidade de vida antes e após o uso do filtro permutador de calor e umidade em laringectomizados totais

**DOI:** 10.1590/2317-1782/e20240282pt

**Authors:** Romulo da Silva Rodrigues, Victória Paes Leme da Silva, Leticia Cristina de Jesus dos Santos, Guilherme Maia Zica, Mariana Pinheiro Brendim

**Affiliations:** 1 Departamento de Fonoaudiologia, Universidade Federal do Rio de Janeiro – UFRJ - Rio de Janeiro (RJ), Brasil.; 2 Programa de Pós-graduação em Distúrbios da Comunicação Humana, Universidade Federal de São Paulo – UNIFESP - São Paulo (SP), Brasil.

**Keywords:** Laringectomia, Traqueostomia, Qualidade de Vida, Respiração, Tosse, Voz, Deglutição, Qualidade do Sono, Patologia da Fala e Linguagem

## Abstract

**Objetivo:**

Comparar a autoavaliação da desvantagem vocal, da qualidade do sono e da qualidade de vida relacionadas à tosse e à deglutição antes e após o uso do filtro permutador de calor e umidade em laringectomizados totais.

**Método:**

Estudo prospectivo longitudinal, no qual os participantes foram submetidos à aplicação dos instrumentos Índice de Desvantagem Vocal (IDV), Índice de Qualidade do Sono de Pittsburgh (PSQI), Leicester Cough Questionnaire (LCQ) e Inventário de Disfagia MD. Anderson (MDADI) em três momentos: (T1) antes do início do uso do filtro permutador de calor e umidade, (T2) após duas semanas e (T3) após quatro semanas de uso do dispositivo.

**Resultados:**

14 participantes (12 homens), com média de idade de 66,4±5,8 anos. As medianas do escore total do IDV em T1, T2 e T3 foram, respectivamente, 65,5(47,5-86,3), 55,5(39,5-71,3) e 44,5(39-72), p=0,085. As medianas do escore do PSQI em T1, T2 e T3 foram, respectivamente, 6,5(4,25-11,8), 4,5(2,25-10,8) e 3,0(2,0-5,75), p=0,010. As medianas do escore total do MDADI em T1, T2 e T3 foram, respectivamente, 78,6(69,1-92,7), 76,3(73,3-92,6) e 85,7(72,7-94), p=0,571. As medianas do escore total do LCQ em T1, T2 e T3 foram, respectivamente, 16,7(13,1-18,5), 19,1(17,4-19,4) e 19,0(17,3-19,9), p=0,002.

**Conclusão:**

Os laringectomizados totais participantes deste estudo apresentam percepção de melhora do domínio emocional da autoavaliação da desvantagem vocal, da qualidade do sono e da qualidade de vida relacionada à tosse após duas semanas de uso de filtro permutador de calor e umidade. Ademais, estes participantes mantêm a percepção de melhora após quatro semanas de uso do dispositivo.

## INTRODUÇÃO

A laringectomia total (LT) é considerada a modalidade primária de tratamento para o carcinoma avançado de laringe, sendo um procedimento cirúrgico agressivo com resultados funcionais e estéticos negativos e duradouros^(1-3)^. Portanto, a literatura evidencia grande impacto na qualidade de vida do paciente devido ao prejuízo e modificações de funções essenciais aos indivíduos: voz, deglutição, olfato, paladar e respiração^(1)^.

As mudanças fisiológicas após LT ocorrem devido à retirada de diversas estruturas, separação das vias respiratórias e digestivas e a construção de um estoma permanente. As modificações específicas na voz e no sistema respiratório são justificadas pela dissociação das vias aéreas superior e inferior, impedindo a produção natural da voz laríngea e interrompendo o caminho normal de preparação do ar pelo nariz^(4-6)^. Ainda sabe-se pouco sobre o processo de deglutição após a LT total. Em estudos manométricos da deglutição, observa-se o aumento da duração da pressão velofaríngea e a diminuição da pressão do esfíncter esofágico superior, o que evidencia os efeitos da ruptura das fibras musculares esofágicas cricofaríngea e rostral de seus anexos para a laringe e a realização da miotomia cricofaríngea^(6)^.

O estoma permite a passagem de fluxo aéreo não condicionado diretamente para as vias aéreas inferiores (traqueia), portanto, impede a manutenção do aquecimento, umidificação, filtração e da resistência fisiológica do ar inalado, além de gerar um déficit na função olfativa. A entrada de ar frio e seco, microrganismos e poeira diretamente nas vias aéreas inferiores aumenta a incidência de danos e infecções broncopulmonares e diminuição da qualidade de vida (QV)^(1,4,7)^.

Devido à história pregressa de tabagismo presente na maioria dos pacientes laringectomizados totais, o estado pós-laringectomia pode apresentar alterações na função pulmonar e prejuízo na ventilação (trocas gasosas). O paciente traqueostomizado tem redução aerodinâmica na resistência ao fluxo aéreo durante a inspiração e a expiração, devido a inalação direta pelo estoma, isso pode ocasionar um efeito negativo na ventilação periférica do pulmão e favorecer o colapso alveolar^(8,9)^.

Um dos mais importantes fatores prognósticos na sobrevida dos laringectomizados é a deterioração progressiva da função pulmonar^(10,11)^. Apesar disso, poucos trabalhos na literatura avaliaram a função pulmonar e a integridade de sua função em pacientes laringectomizados e/ou descrevem a reabilitação e seus efeitos fazendo uso de metodologia reprodutível^(4,11-14)^. De acordo com um estudo, a maioria dos pacientes submetidos à LT apresenta função pulmonar alterada, do tipo obstrutiva, quase sempre associada à história de tabagismo^(11)^.

O conceito de QV é complexo, multifacetado e dependente da visão do indivíduo, no que diz respeito não apenas à saúde física, psicológica e funcional, mas também ao bem-estar social e financeiro^(15-17)^. A cirurgia de LT é desfigurante e garante um estoma permanente, o que é potencialmente estigmatizante e altera a comunicação e os relacionamentos íntimos, com complicações de difícil gerenciamento como o excesso de secreção pulmonar, tosse recorrente e dificuldades no sono^(1,4,7,12,15)^. Além disso, a integração social pós-operatória tem se mostrado deficitária, com depressão, ansiedade e autoisolamento^(1,18)^. Estes aspectos reafirmam a necessidade de investigação multidimensional da percepção individual dos sujeitos, sua posição na vida, no contexto da cultura e dos sistemas de valores em que vivem e seus objetivos, expectativas, padrões e preocupações.

Diferentes queixas respiratórias apresentam importantes correlações com problemas físicos e psicossociais dos indivíduos. Normalmente a reabilitação apresenta-se com maior foco nos aspectos de voz e, por vezes, deglutição e olfato^(5,6)^. Deste modo, acredita-se que um programa de reabilitação fonoaudiológica completo para o paciente laringectomizado total deve considerar a gerência dos déficits respiratórios decorrentes do procedimento cirúrgico^(4,19)^.

Uma opção viável para o processo de reabilitação pulmonar após a LT é o *heat and moisture exchanger* (HME), um dispositivo permutador de calor e umidade colocado sobre uma vedação hermética em volta do estoma traqueal. O HME tem três propriedades físicas: capacidade de troca de calor e umidade, adição de resistência ao fluxo de ar e filtragem de partículas compatível com a função do nariz. Existem diferentes adesivos (vedação do estoma e base de fixação), com propriedades de aderência e adaptação distintas para viabilizar a funcionalidade a diferentes pacientes e suas variações anatômicas de cada estoma^(4,12-14)^.

Na experiência clínica e em diferentes estudos, é perceptível a redução das queixas de tosse e produção de muco em usuários do HME laringectomizados totais^(4,19)^. No entanto, principalmente no Brasil e América Latina, as evidências científicas que comprovem os benefícios desse dispositivo e a importância do seu uso ainda são restritas.

Sendo assim, o objetivo deste estudo é comparar a autoavaliação da desvantagem vocal, da qualidade do sono e da qualidade de vida relacionadas à tosse e à deglutição antes e após o uso do filtro permutador de calor e umidade em indivíduos laringectomizados totais de um hospital universitário no Brasil.

## MÉTODO

Este estudo foi aprovado pelo Comitê de Ética em Pesquisa do Hospital Universitário Clementino Fraga Filho (HUCFF), sob o parecer 3.442.414. Todos os participantes assinaram o Termo de Consentimento Livre e Esclarecido.

Trata-se de um ensaio não controlado, realizado entre agosto de 2019 e maio de 2022 no Ambulatório de Fonoaudiologia do HUCFF. Os critérios de inclusão foram indivíduos adultos, submetidos ao procedimento cirúrgico de LT e em acompanhamento fonoaudiológico neste hospital. Os critérios de exclusão foram: indivíduos com doenças neurológicas, com alterações cognitivas ou de linguagem, em cuidados de fim de vida e que já utilizavam o filtro umidificador de traqueostomia.

Os participantes foram recrutados consecutivamente, sendo incluídos todos os indivíduos laringectomizados totais em acompanhamento fonoaudiológico neste hospital no período de realização do estudo. A amostra foi constituída por 16 homens e duas mulheres submetidos à cirurgia de LT, com média de idade de 66,2 ± 5,3 anos.

Indivíduos que iniciaram o estudo e interromperam por qualquer motivo o acompanhamento durante o período de realização dos procedimentos do estudo foram considerados como perda de seguimento. Sendo assim, permaneceram no estudo somente aqueles que realizaram todas as etapas do estudo sem interrupção.

Todos os participantes foram submetidos à aplicação da versão brasileira dos questionários Índice de Desvantagem Vocal (IDV)^(20)^, Inventário de Disfagia MD. Anderson (MDADI)^(21)^, *Leicester Cough Questionnaire* (LCQ)^(22)^ e Índice da Qualidade do Sono de Pittsburgh (PSQI)^(23)^ em três momentos: (T1) antes do início do uso do filtro permutador de calor e umidade, (T2) após duas semanas de uso do filtro permutador de calor e umidade e (T3) após quatro semanas de uso do filtro permutador de calor e umidade.

O IDV^(24)^, adaptado e validado para o português brasileiro, consiste em 30 itens, cujas opções de resposta abarcam três domínios: funcional, orgânico e emocional^(20)^. A pontuação de cada domínio varia entre 0 e 40 e do escore total entre 0 e 120 pontos. Quanto maior a pontuação, maior é a percepção de desvantagem vocal^(20)^.

O MDADI^(25)^, adaptado e validado para o português brasileiro, permite a avaliação da qualidade de vida relacionada à disfagia especificamente para a população com câncer de cabeça e pescoço^(21)^. Ele consiste em 20 itens que abrangem a questão global e os domínios físico, funcional e emocional, com variação até 100 pontos. Quanto menor a pontuação, maior é o impacto na qualidade de vida^(21)^.

O LCQ^(26)^, traduzido e adaptado para o português brasileiro, permite a avaliação da qualidade de vida em indivíduos portadores de tosse. Ele consiste em 19 itens, compreendendo os domínios físico, psicológico e social. A pontuação de cada domínio varia entre um e sete pontos e o escore total entre três e 21, sendo escores próximos de 21 indicativos de melhor estado de saúde ou menor influência da tosse na qualidade de vida^(22)^.

O PSQI^(27)^, traduzido e validado para o português brasileiro, permite a avaliação da qualidade e distúrbios do sono. A pontuação deste instrumento varia até 21 pontos, sendo que pontuações acima de cinco indicam má qualidade do sono^(23)^.

Além disso, foram coletadas informações sociodemográficas e clínicas dos participantes, tais como: gênero; idade; tempo de laringectomia, que compreendeu o período entre a data da cirurgia e a data de entrada no estudo; se realizou esvaziamento cervical, radioterapia ou quimioterapia para o tratamento da doença; presença ou ausência de doença pulmonar e de cânula de traqueostomia; se realizou traqueostomia de emergência (antes da cirurgia) ou no momento da LT; história de tabagismo; e método de comunicação alaríngea utilizado.

Em relação à adaptação do filtro permutador de calor e umidade, foram realizados encontros semanais no ambulatório de Fonoaudiologia do HUCFF para adaptação do dispositivo. Durante quatro semanas consecutivas, os participantes receberam semanalmente sete filtros Provox^®^ XtraFlow^TM^ HME e sete adesivos Provox^®^ Adhesive OptiDerm^TM^ oval. Os participantes foram orientados a realizar a higiene local, em seguida, aderir o adesivo sobre a pele limpa na altura do traqueostoma e, então, adaptar o filtro no suporte do adesivo. Os participantes que utilizavam cânula de traqueostomia foram orientados a adaptar o filtro na cânula Provox^®^ LaryTube^TM^. Todos os participantes foram orientados a utilizar o filtro em tempo integral, retirando apenas para a realização da troca por outro filtro a cada 24 horas ou para higienização da cânula de traqueostomia.

Durante as quatro semanas de uso do dispositivo, os participantes não foram submetidos à terapia fonoaudiológica de deglutição, drenagem linfática manual ou liberação miofascial. Este estudo não empregou a orientação de uso de inalação. Além disso, nenhum participante realizava fisioterapia respiratória.

A análise estatística foi realizada através do programa Jamovi versão 1.6.23. A normalidade dos dados foi verificada através do histograma. Para comparação da autoavaliação antes e após o uso do filtro umidificador de traqueostomia, foram comparados os escores apresentados em T1, T2 e T3. Os escores dos questionários foram comparados através do teste ANOVA de Friedman, seguido pelo teste de comparação múltipla de Durbin-Conover. O nível de significância estatística adotado foi de 5%.

## RESULTADOS

O fluxograma dos participantes do estudo é descrito na Figura 1. Dos 18 participantes incluídos no estudo, dois foram excluídos e dois apresentaram perda de seguimento, totalizando 14 participantes na amostra final.

**Figura 1:** Fluxograma dos participantes do estudo

A amostra final compreendeu 12 homens e duas mulheres, com idade entre 60 e 81 anos e média de 66,4±5,8 anos. As características dos participantes são apresentadas na Tabela 1. Os participantes com doença pulmonar apresentavam doença pulmonar obstrutiva crônica. Quanto ao método de comunicação, dois (14,3%) participantes utilizavam a voz traqueoesofágica e 12 (85,7%) utilizavam a voz esofágica, sendo quatro participantes fluentes e os demais em aprendizado.

**Tabela 1:** Características dos participantes

**Características**	**Total (n=14)**
Gênero masculino, n (%)	12 (85,7%)
Idade (anos), mediana (IIQ)	66 (61,5-69)
Tempo de laringectomia (meses), mediana (IIQ)	15 (8,5-29,3)
Esvaziamento cervical, n (%)	13 (92,9%)
Radioterapia, n (%)	12 (85,7%)
Quimioterapia, n (%)	6 (42,9%)
História de tabagismo, n (%)	12 (85,7%)
Doença pulmonar, n (%)	4 (28,6%)
Realização de TQT de urgência, n (%)	5 (35,7%)
Utilização da cânula de TQT *(LaryTube)*	1 (7,1%)

Legenda: IIQ = Intervalo interquartílico; TQT = traqueostomia

As comparações do escore total e dos domínios físico, orgânico e emocional do IDV são apresentadas na Tabela 2. Houve diferença estatisticamente significativa dos escores do domínio emocional (*x* ^2^ (2) = 10,1, p = 0,006). A análise post hoc indicou diferenças estatisticamente significativas dos escores do domínio emocional entre T1 e T2 (p=0,022) e T1 e T3 (p=0,001), mas não entre T2 e T3 (p=0,192).

**Tabela 2:** Comparação do IDV entre T1, T2 e T3

**IDV**	**T1**	**T2**	**T3**	**p valor**
Domínio físico	27(21,3-30,8)	24,5(21,3-30,8)	25(16,5-30,3)	0,651
Domínio orgânico	20,5(11,3-24,5)	15(8,75-23,3)	12,5(9,5-20,8)	0,614
Domínio emocional	22,5(7,25-32)	14,5(5,75-23,5)^a^	14(4,5-18,8)^b^	**0,006**
Escore total	65,5(47,5-86,3)	55,5(39,5-71,3)	44,5(39-72)	0,085

Teste de Friedman. Comparação por pares pelo teste de Durbin-Conover:

a p=0,022 na comparação entre T1 e T2;

b p< 0,001 na comparação entre T1 e T3

Valores apresentados em mediana e intervalos interquartílicos

Legenda: IDV = Índice de desvantagem vocal; T1 = Avaliação inicial; T2 = Avaliação após duas semanas; T3 = Avaliação após quatro semanas

As comparações dos escores totais, da questão global e dos domínios emocional, funcional e físico do MDADI são apresentadas na Tabela 3. Não houve diferença estatisticamente significativa de nenhum dos escores do MDADI.

**Tabela 3:** Comparação do MDADI entre T1, T2 e T3

**MDADI**	**T1**	**T2**	**T3**	**p valor**
Questão global	70(40-80)	80(45-100)	100(50-100)	0,087
Domínio emocional	80(73,3-100)	81,7(69,2-100)	91,7(77,5-100)	0,590
Domínio funcional	84(80-95)	86(80-96)	94(78-96)	0,832
Domínio físico	71,3(65-80)	76,3(65-81,9)	68,8(65-84,4)	0,853
Escore total	78,6(69,1-92,7)	76,3(73,3-92,6)	85,7(72,7-94,0)	0,571

Teste de Friedman

Valores apresentados em mediana e intervalos interquartílicos

Legenda: MDADI = Inventário de disfagia MD. Anderson; T1 = Avaliação inicial; T2 = Avaliação após duas semanas; T3 = Avaliação após quatro semanas

As comparações do escore total e dos domínios físico, psicológico e social do LCQ são apresentadas na Tabela 4. Houve diferenças estatisticamente significativas nos escores totais (*x* ^2^ (2) = 12,7, p = 0,002), do domínio físico (*x* ^2^ (2) = 9,16, p = 0,010), do domínio psicológico (*x* ^2^ (2) = 10,1, p = 0,006) e do domínio social (*x* ^2^ (2) = 9,94, p = 0,007) do LCQ entre os tempos de avaliação. A análise post hoc indicou diferenças estatisticamente significativas dos escores totais (p = 0,003) e dos domínios físico (p = 0,032) e psicológico (p = 0,016) do LCQ entre T1 e T2. Além disso, a análise post hoc indicou diferenças estatisticamente significativas dos escores totais (p < 0,001) e de todos os domínios (físico, p = 0,039; psicológico, p = 0,004; e social, p = 0,02) entre T1 e T3. Não houve diferença estatisticamente significativa de nenhum escore entre T2 e T3.

**Tabela 4:** Comparação do LCQ entre T1, T2 e T3

**LCQ**	**T1**	**T2**	**T3**	**p valor**
Domínio físico	4,88(3,81-5,60)	6,13(5,44-6,25)^a^	6,13(5,25-6,44)^b^	**0,010**
Domínio psicológico	5,29(4,33-6,10)	6,14(5,43-6,57)^a^	6,14(5,82-6,60)^b^	**0,006**
Domínio social	6,25(5,31-7,00)	6,88(6,19-7,00)	7,00(6,19-7,00)^b^	**0,007**
Escore total	16,7(13,1-18,5)	19,1(17,4-19,4)^a^	19,0(17,3-19,9)^b^	**0,002**

Teste de Friedman. Comparação por pares pelo teste de Durbin-Conover:

a p < 0,05 na comparação entre T1 e T2;

b p < 0,05 na comparação entre T1 e T3

Valores apresentados em mediana e intervalos interquartílicos

Legenda: LCQ = Questionário de tosse de Leicester; T1 = Avaliação inicial; T2 = Avaliação após duas semanas; T3 = Avaliação após quatro semanas

As comparações dos escores dos componentes do PSQI são apresentadas na Tabela 5. Houve diferença estatisticamente significativa do escore total do PSQI entre os tempos de avaliação (*x* ^2^ (2) = 9,23, p = 0,010). A análise post hoc indicou diferenças estatisticamente significativas dos escores entre T1 e T2 (p = 0,029) e T1 e T3 (p = 0,002), mas não entre T2 e T3 (p = 0,236). Além disso, houve diferença estatisticamente significativa dos escores do componente de alterações do sono entre os tempos de avaliação (*x* ^2^ (2) = 9,00, p = 0,011). A análise post hoc indicou diferenças estatisticamente significativas dos escores deste componente entre T1 e T3 (p = 0,002), mas não entre T1 e T2 (p = 0,091) e entre T2 e T3 (p = 0,091).

**Tabela 5:** Comparação da qualidade do sono entre T1, T2 e T3

**Componentes do PSQI**	**T1**	**T2**	**T3**	**p valor**
Qualidade subjetiva do sono	1(1-2)	1(0-1,75)	1(0-1)	0,054
Latência do sono	1(1-1)	1(1-1,75)	1(0-1)	0,661
Duração do sono	1(0,25-1)	0,5(0-2)	0(0-1)	0,069
Eficiência habitual do sono	1 (0-2)	1(0-2)	0(0-1,75)	0,459
Alterações do sono	2(1-2)	1(1-2)	1(1-1)^b^	**0,011**
Uso de medicação para o sono	0(0-0,75)	0(0-0)	0(0-0)	0,150
Disfunção diurna	1(0-1,75)	0(0-1)	0(0-1)	0,597
Escore total	6,50(4,25-11,8)	4,50(2,25-10,8)^a^	3(2,0-5,75)^b^	**0,010**

Teste de Friedman. Comparação por pares pelo teste de Durbin-Conover:

a p < 0,05 na comparação entre T1 e T2;

b p < 0,05 na comparação entre T1 e T3

Valores apresentados em mediana e intervalos interquartílicos

Legenda: PSQI = Índice da qualidade do sono de Pittsburgh

## DISCUSSÃO

Este parece ser o primeiro estudo que investigou, por meio de instrumentos de autoavaliação validados, a percepção de laringectomizados totais brasileiros sobre os aspectos vocais, da deglutição e do sono, antes e após o uso do filtro permutador de calor e umidade. Além disso, este estudo investigou a autoavaliação da qualidade de vida relacionada à tosse, por meio de um instrumento traduzido e adaptado para o português brasileiro, antes e após o uso do filtro permutador de calor e umidade. Os resultados do presente estudo evidenciam, de forma geral, melhora de aspectos relacionados à percepção de desvantagem vocal, da qualidade de vida relacionada à tosse e da qualidade do sono após o uso do filtro permutador de calor e umidade.

Em consonância com a literatura, o perfil dos participantes do presente estudo foi constituído predominantemente por indivíduos idosos, do gênero masculino e com história de tabagismo, já que o câncer de laringe é mais frequente em homens, com média de idade acima de 60 anos, sendo o tabagismo o seu principal fator de risco^(28-30)^.

Em relação à percepção de desvantagem vocal, a mediana do escore total do IDV encontrado neste estudo, cuja maioria dos participantes utilizava voz esofágica, foi semelhante à média do escore total do IDV apontada por outros pesquisadores em indivíduos com voz esofágica^(31)^.

Apesar dos nossos resultados revelarem uma redução do escore total e dos escores dos domínios físico, orgânico e emocional do IDV ao longo das quatro semanas de uso do filtro permutador de calor e umidade, apenas o domínio emocional apresentou uma mudança estatisticamente significativa tanto em duas semanas quanto em quatro semanas de uso do filtro. Isso significa que há uma percepção de melhora relacionada aos aspectos emocionais a partir de duas semanas de uso do filtro permutador de calor e umidade e que essa percepção de melhora se mantém após quatro semanas de uso do dispositivo. O domínio emocional deste instrumento abrange itens relacionados à frustração em situações de comunicação geral, bem como a percepção de desvantagem gerada pela alteração vocal, constrangimento ao repetir enunciados, sensação de incompetência e vergonha^(32)^.

Ao encontro dos nossos resultados, um estudo, que avaliou a resposta de laringectomizados totais brasileiros à pergunta “o que você acha da sua voz?”, revelou melhora da autopercepção vocal em 70% dos indivíduos após duas semanas de uso do filtro permutador de calor e umidade^(4)^. Em contrapartida, outro estudo que investigou a avaliação perceptivo-auditiva da voz de laringectomizados brasileiros não encontrou influência do uso do filtro permutador de calor e umidade por seis semanas na qualidade vocal esofágica ou traqueoesofágica^(12)^.

Quanto à qualidade de vida relacionada à deglutição, os escores do MDADI encontrados nos participantes deste estudo foram semelhantes aos divulgados na literatura^(28,29)^. Além disso, de acordo com a proposta de Chen et al.^(33)^, a mediana do escore total do MDADI revelada neste estudo indica uma limitação média na qualidade de vida em deglutição desses indivíduos.

Este estudo não evidenciou nenhuma mudança estatisticamente significativa dos escores da questão global, dos domínios emocional, funcional e físico, bem como do escore total do MDADI após o uso do filtro permutador de calor e umidade, o que indica que não há uma percepção de melhora nem de piora da qualidade de vida em deglutição pelos participantes deste estudo, seja após duas ou quatro semanas de uso do dispositivo. Acredita-se que a deglutição em pacientes submetidos à LT precisa ter uma intervenção focada e especializada para controle e superação de déficits motores e psicoemocionais.

Os resultados deste estudo evidenciam que indivíduos laringectomizados totais exibem melhora de todos os domínios da qualidade de vida relacionada à tosse após quatro semanas de uso do filtro permutador de calor e umidade. Após a cirurgia, em virtude das mudanças anatômicas e redirecionamento do fluxo respiratório via traqueostomia nesses indivíduos, o ar não condicionado, flui diretamente para o epitélio do trato respiratório inferior, podendo causar alterações histológicas na mucosa traqueobrônquica, produção excessiva de secreção, tosse involuntária recorrente e expectoração forçada para desobstruir as vias aéreas do muco^(34,35)^. Sendo assim, os resultados referentes à melhora da qualidade de vida relacionada à tosse neste estudo podem ser justificados pela ação do dispositivo de filtragem, aquecimento e umidificação do ar inalado via traqueostomia. Após duas semanas de uso do filtro permutador de calor e umidade já é possível observar percepção de melhora significativa nos domínios físico e psicológico, além do escore total, mas não do domínio social da qualidade de vida. Os resultados estão em concordância com outros estudos, que também encontraram melhora na autopercepção da tosse, na frequência de tosse e no índice de qualidade de vida geral após duas semanas de uso do filtro permutador de calor e umidade^(4,13)^.

Uma possível explicação para o domínio social ter apresentado melhora somente após quatro semanas de uso do filtro seja pelo fato da questão social depender da interação social e da habilidade de fala, que levam mais tempo na reabilitação para serem desenvolvidas em laringectomizados totais que utilizam a voz esofágica. Outros estudos constataram redução do isolamento social e maior frequência de interação social entre aqueles que utilizam o filtro permutador de calor e umidade, devido à maior facilidade de comunicação, melhor convívio social e menor constrangimento relacionado à produção e expectoração de secreções^(18,36)^.

Sobre a qualidade do sono, nossos resultados mostram que, antes do uso do filtro permutador de calor e umidade, a mediana do escore total do PSQI foi maior que cinco, indicando que pelo menos metade dos participantes apresentava má qualidade do sono. Aspecto este, pouco considerado no processo de reabilitação e condução após a cirurgia de LT.

Após duas semanas de uso do filtro, os participantes apresentaram melhora significativa da percepção da qualidade do sono e esta percepção de melhora se mantém após quatro semanas de uso do dispositivo. Um estudo de metanálise evidenciou que o filtro permutador de calor e umidade, em comparação com o umidificador externo, é o dispositivo que acarreta menos problemas do sono após a LT^(37)^. Além disso, as medianas do escore total do PSQI inferiores a cinco após duas e quatro semanas de uso do filtro permutador de calor e umidade indicam que, diferentemente da percepção antes do uso do dispositivo, pelo menos metade dos participantes deste estudo passaram a não apresentar percepção de má qualidade do sono.

A percepção de melhora da qualidade do sono nesses indivíduos pode ser explicada pela possibilidade desses indivíduos exibirem maior conforto ao respirar e menor risco de despertar durante o sono, em virtude da redução da secreção e, consequentemente, da tosse após o uso do dispositivo. Além disso, a segurança de não apresentar um orifício aberto durante a noite pode permitir maior conforto e relaxamento durante o sono.

Ressalta-se que este estudo apresenta algumas limitações importantes. A primeira delas relaciona-se à ausência de um grupo controle (sem utilização do filtro permutador de calor e umidade) para comparação com o grupo submetido ao uso do dispositivo. Portanto, este estudo não permite afirmar que as mudanças observadas são decorrentes do uso do filtro permutador de calor e umidade, já que este não é um ensaio clínico randomizado. A segunda limitação refere-se ao tamanho da amostra, que incluiu um número pequeno de participantes e, assim, a possibilidade de erro tipo II, além da impossibilidade de generalização dos resultados encontrados. Outra limitação relaciona-se aos instrumentos utilizados. O MDADI, apesar de ser um instrumento para avaliação da qualidade de vida relacionada à deglutição validado em nossa língua para a população com câncer de cabeça e pescoço, não foi desenvolvido especificamente para laringectomizados totais^(38)^. Além disso, o instrumento LCQ, apesar de ser um instrumento traduzido e adaptado para o português brasileiro que permite a avaliação da qualidade de vida relacionada à tosse, não concluiu as etapas de validação.

## CONCLUSÃO

Laringectomizados totais participantes deste estudo apresentam percepção de melhora do domínio emocional da autoavaliação da desvantagem vocal, da qualidade do sono e da qualidade de vida relacionada à tosse após duas semanas de uso de filtro permutador de calor e umidade. A percepção de melhora de tais aspectos é mantida após quatro semanas de uso do dispositivo, apesar de não haver percepção de melhora da qualidade de vida em deglutição após duas ou quatro semanas de uso do dispositivo nos participantes deste estudo.
